# Systemic Inflammatory Symptoms Following Mastectomy With Nickel-Containing Surgical Clips: A Case Report

**DOI:** 10.7759/cureus.116785

**Published:** 2026-09-23

**Authors:** Jasmin Kaur, Anna Kokesh, Frederick Tiesenga

**Affiliations:** 1 Medicine, Windsor University School of Medicine, Cayon, KNA; 2 Medicine, Saint James School of Medicine, Arnos Vale, VCT; 3 General Surgery, West Suburban Medical Center, Oak Park, USA

**Keywords:** allergic contact dermatitis (acd), hypersensitivity, implant, metal allergy, nickel hypersensitivity, prophylactic bilateral mastectomy, surgery, surgical clips, systemic immune inflammation, titanium

## Abstract

Systemic inflammatory symptoms, such as metal hypersensitivity to surgical materials, are uncommon but very underrecognized complications that may present with persistent systemic and musculoskeletal symptoms.

This case involves a 36-year-old female with a BRCA2 mutation who underwent bilateral prophylactic mastectomy with immediate breast implant reconstruction. Approximately four weeks after surgery, she developed bilateral fatigue, hand, foot, and ankle swelling, severe joint pain, and muscle weakness, leading to significant difficulty in performing activities of daily living. She proceeded to pursue multiple integrative therapies. About six years later, chest radiography incidentally revealed retained surgical metallic clips located at the mastectomy sites. The clips were identified as Ethicon T20 titanium clips containing trace amounts of nickel. The patient then recalled a history of allergic contact dermatitis associated with nickel-containing jewelry, and further lymphocyte transformation testing (LTT) reported reactivity to both nickel and iron. Following difficulty locating a surgeon willing to remove the clips, the patient underwent surgical removal of 19 retained clips using intraoperative fluoroscopic guidance.

This case highlights the diagnostic challenges associated with persistent postoperative systemic symptoms and the importance of considering uncommon variables, such as metal hypersensitivity, in patients with unexplained prolonged inflammatory and musculoskeletal symptoms following implantation or placement of surgical materials.

## Introduction

Nickel is a commonly known metal allergen and is known to cause allergic dermatitis, categorized as a delayed-type hypersensitivity reaction. Exposure to nickel can occur through common items such as jewelry, coins, clothing, cosmetics, and medical devices [1]. Contact-related nickel allergy usually presents as an itchy rash, as the ions released from various alloys trigger skin inflammation [1]. Local hypersensitivity reactions may be easily identified, whereas systemic manifestations are less recognized and may be difficult to distinguish from infectious, autoimmune, or inflammatory disorders.

Surgical clips are sterile medical instruments frequently used during surgery to mark surgical anatomy, approximate tissues, and achieve hemostasis [2]. These clips are usually made of high-grade titanium with trace components of other metals, such as nickel and other alloys. While they are used during medical procedures because of their effectiveness in providing a quicker, less invasive method and enhancing healing and surgical safety, surgical clips may pose rare risks [2]. Although surgical clip placement is generally well tolerated, their metallic composition may be clinically relevant and pose a possible concern in patients with known or suspected hypersensitivity.

Patients who develop unexplained systemic symptoms following a procedure or surgery may undergo an extensive diagnostic evaluation to identify the underlying etiology before the contribution of a surgically implanted material or device is considered. Common and nonspecific symptoms, such as joint pain, muscle weakness, peripheral edema, and skin changes, may lead to evaluation for infectious, dermatologic, or rheumatologic disease.

## Case presentation

A 36-year-old Caucasian female with a significant family history of BRCA2 gene-associated breast cancer had undergone genetic testing after her mother and two sisters prophylactically underwent BRCA2 testing, since her maternal grandmother had a history of BRCA-associated cancer. While the genetic testing results found the two sisters negative, the patient was found to be a carrier of the BRCA2 mutation.

Six years prior to presentation, she decided to undergo prophylactic breast surgery and underwent bilateral mastectomy and immediate breast reconstruction with implant placement during the same operative procedure. She reported that the surgery was unremarkable, without complications. Without the patient’s knowledge, numerous hemostatic surgical clips were permanently placed during the procedure.

Approximately four weeks postoperatively, the patient developed constant pain, which worsened in the morning, and swelling involving the fingers of both hands, which she described as resembling “scleroderma.” The symptoms were initially present bilaterally in the upper extremities and subsequently became more prominent in the left fingers (Figure 1). Similar symptoms subsequently developed in both feet, with involvement extending to both ankles and knees (Figure 2). During this time, the patient’s husband reported that he went from being married to a “30-year-old running three miles/5 days a week” to now caring for what he felt was a “90-year-old,” representing a profound deterioration in physical functioning. The patient reported that she could not use her hands to grip anything. For example, she would have to use the palms of both hands to pick up and hold a water bottle. She also reported generalized fatigue. She could start one load of laundry and then have to take a break. The patient was subsequently told that her symptoms could possibly represent post-COVID rheumatoid arthritis. Further evaluation revealed elevated viral loads for cytomegalovirus (CMV), Epstein-Barr virus (EBV), and human herpesvirus 6 (HHV-6); however, subsequent autoimmune testing was negative. She therefore continued to explore other potential explanations for her symptoms and sought natural approaches to symptom management.

**Figure 1:**
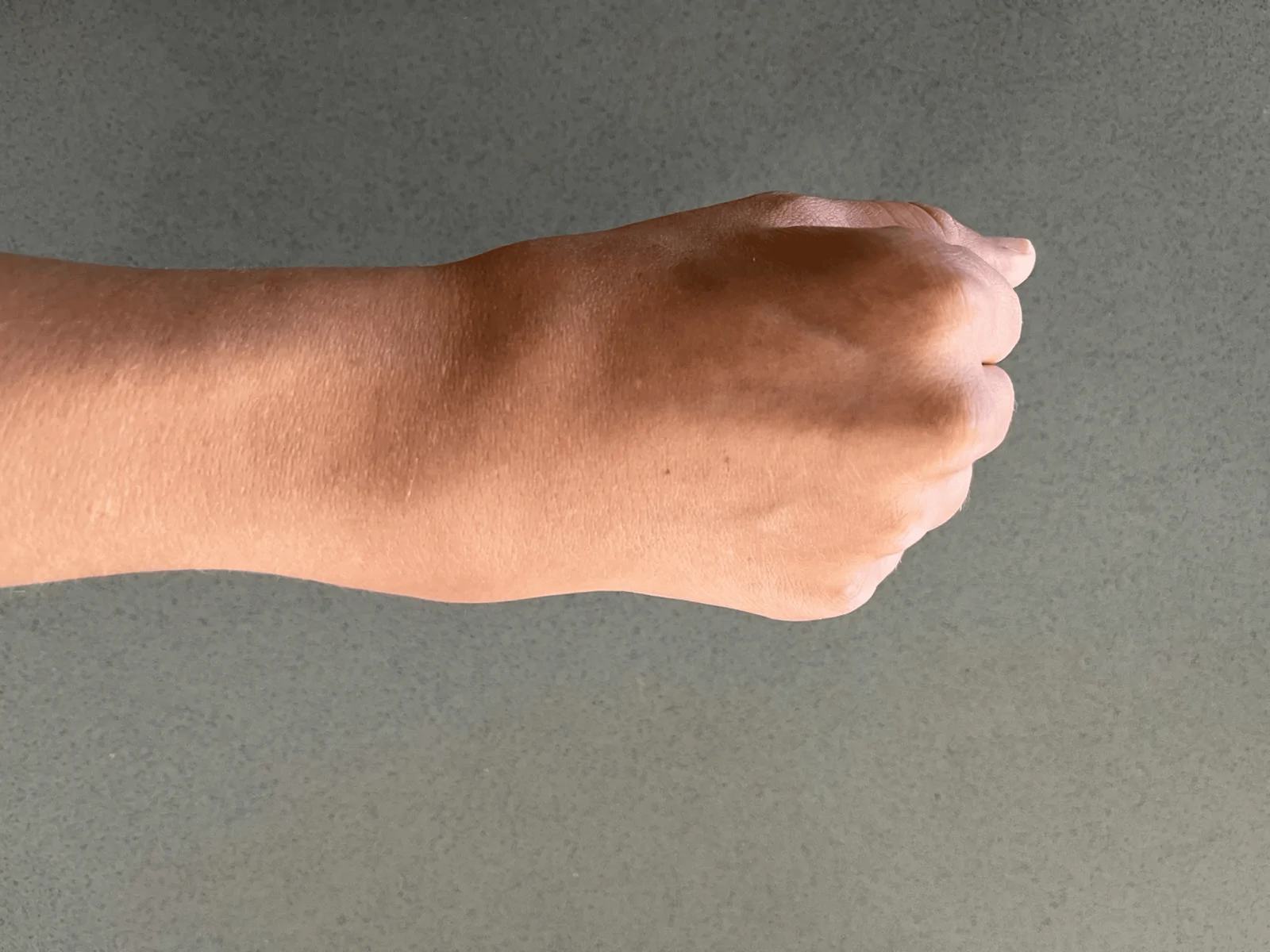
Right wrist swelling following surgical clip placement

**Figure 2:**
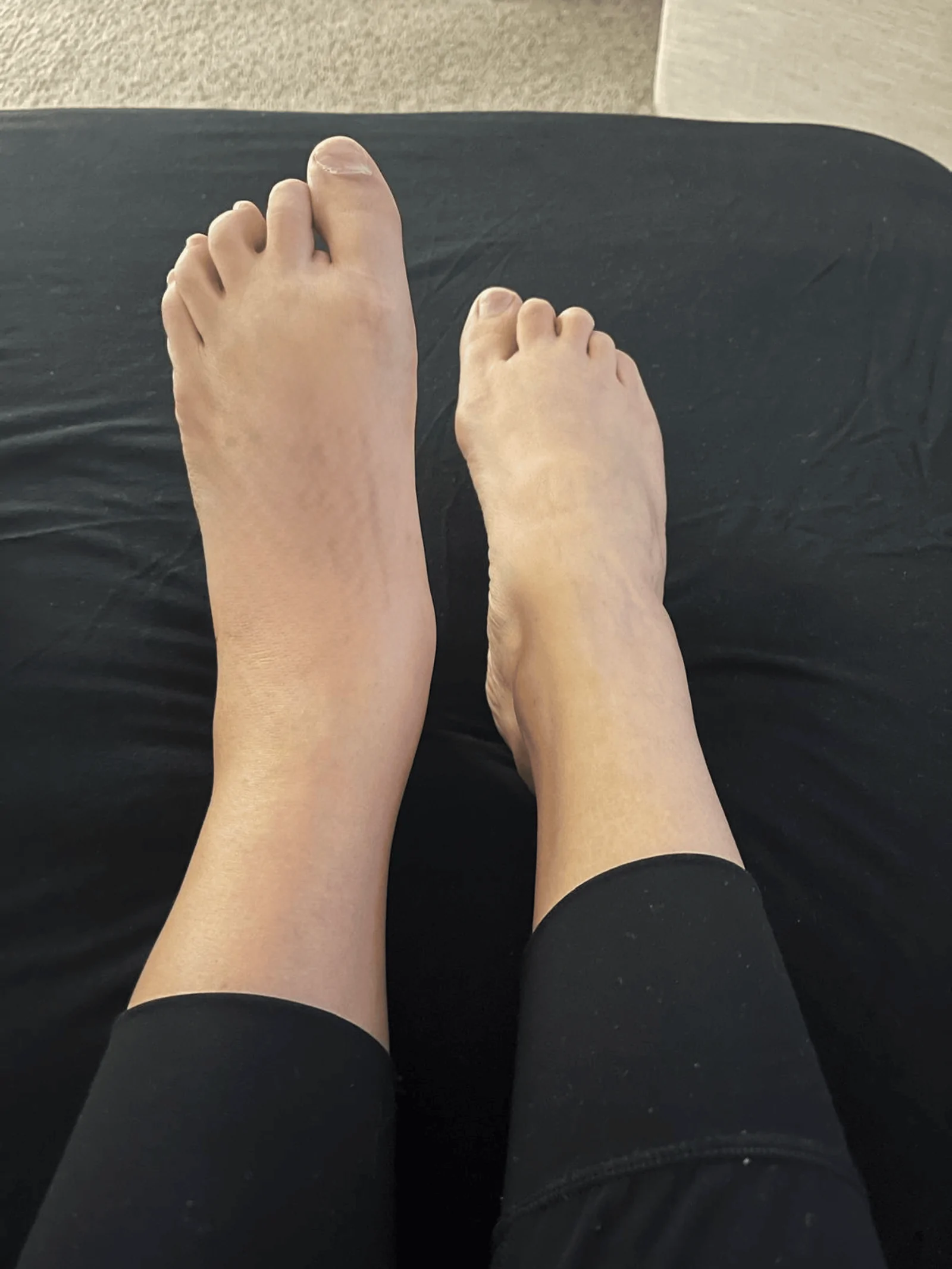
Peripheral swelling of bilateral lower extremities

The patient looked locally and traveled nationally for various treatments, including various detoxifications, supplements, IV ozone therapy, “Extracorporeal Blood Oxygenation and Ozonation” therapy, infrared sauna, removal of the metal fillings from her teeth, removal of her breast implants (no surgical clips were placed during this surgery), chiropractic therapy, discontinuation of conventional deodorants, laundry detergents, and cosmetics, and “Plasma Rich Fibrin” treatments injected into the joints of her hands. After these various treatments, she reported that they helped to “maintain her pain.” She did not feel like she was in “constant fight or flight anymore.” However, despite continuing these therapies for five years, she still experienced persistent pain, swelling, and functional impairment in her joints.

Approximately six years after the initial procedure, the patient underwent chest radiography performed by her chiropractor, during which metallic foreign bodies were incidentally identified within both sides of her chest and axillary areas. She then reached out to her breast surgeon and discovered that these were placed during her mastectomy surgery for blood loss control and was told many times that these clips “are safe.” The clips were identified as Ethicon T20 titanium clips, which the patient reported contained trace amounts of nickel based on her review of product information and composition. She subsequently investigated the composition of the surgical clips and reported finding product information indicating that the material contained trace amounts of nickel. Prior to her double mastectomy, the patient did not report an allergy to nickel. It was not until after the surgery that she recalled that she could not wear nickel jewelry due to allergic contact dermatitis symptoms.

With this new information, the patient subsequently underwent lymphocyte transformation testing (LTT), which demonstrated lymphocytic reactivity to nickel and iron. Based on these results and her persistent symptoms, she decided to undergo surgical removal of the metal clips.

The patient reported difficulty finding a surgeon who was willing to remove her retained surgical clips. She contacted 13 surgeons regarding clip removal; however, she was informed that they would not remove the clips. Only one surgeon was willing to remove the clips closer to the chest but still did not want to remove the clips in the axillary area. While continuing to search for information regarding surgical clip removal, she began researching medical devices and related resources and came across a podcast featuring individuals who reported experiencing symptoms similar to hers following placement of surgical clips. She subsequently contacted one of the individuals featured on the podcast, who provided her with the name of the surgeon who had previously removed that individual’s surgical clips. This led her to Dr. Frederick Tiesenga in Chicago, Illinois. Approximately one month later, the patient underwent surgical removal of retained surgical clips from the mastectomy sites, with intraoperative fluoroscopic imaging used for localization and guidance during removal (Figure 3). Nineteen clips were removed during this operation (Figure 4).

**Figure 3:**
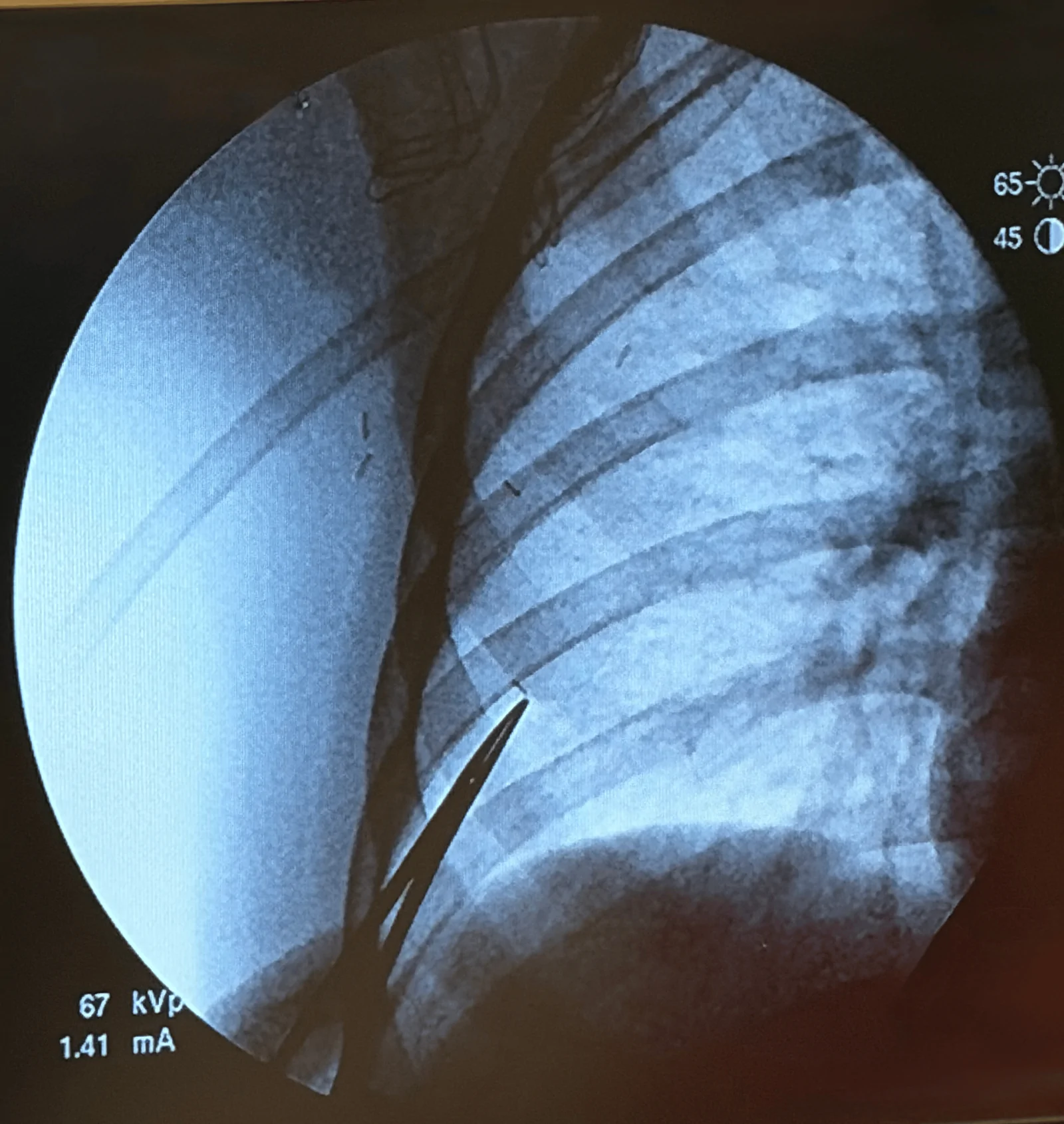
Intraoperative fluoroscopic imaging showing localization and real-time guidance during successful removal of the metallic surgical clips

**Figure 4:**
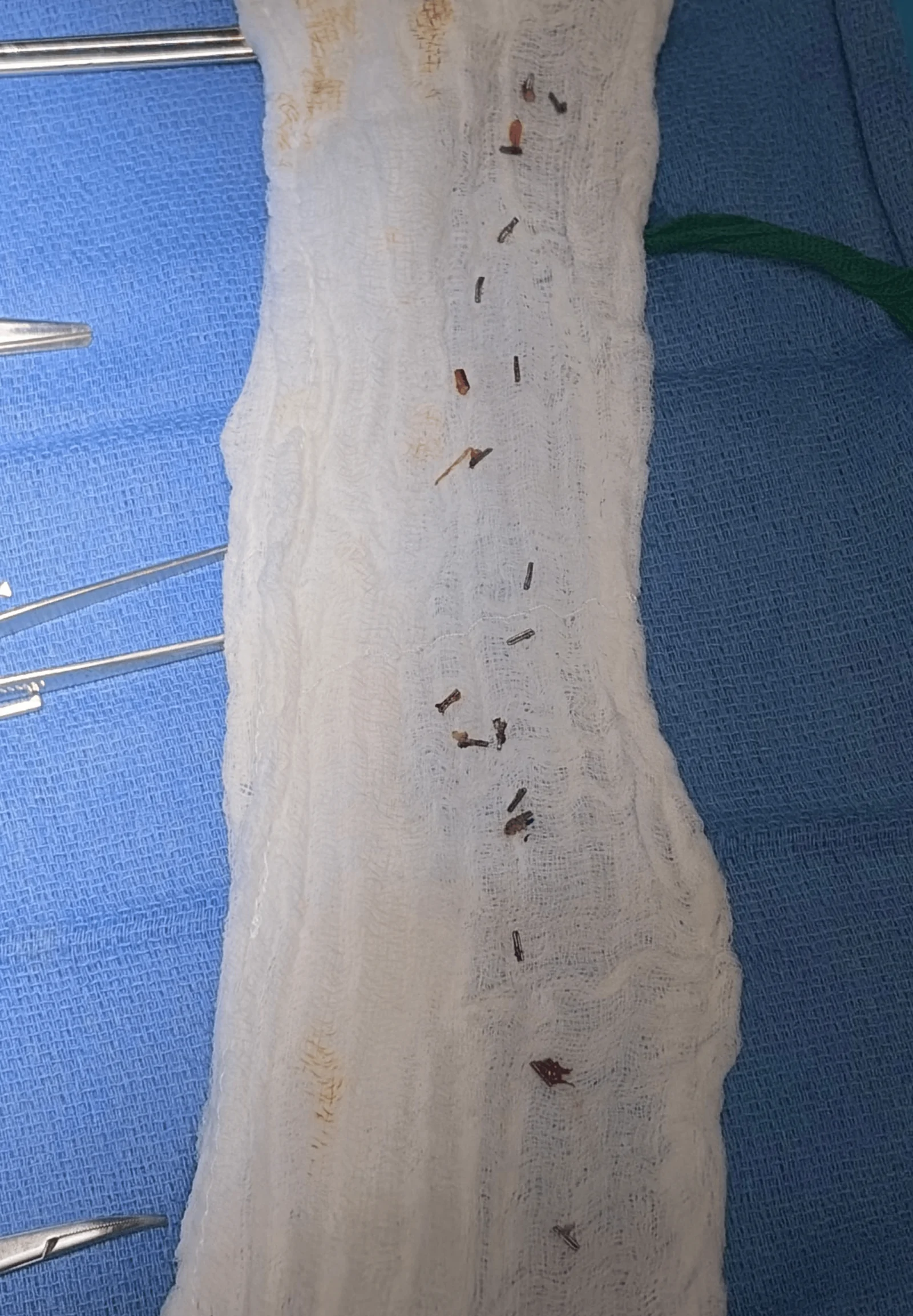
Retained surgical clips following removal

At the 13-day postoperative follow-up, following removal of the metal clips, the patient reported a marked improvement in her symptoms. She described a substantial improvement in wrist swelling, noting that the bony prominences of both wrists, which had previously been obscured by swelling, were now visibly apparent (Figure 5). She also reported improved wrist function and an increased ability to use her wrists for daily activities. The patient reported that her pain had been reduced and was no longer “waking up in pain and going to bed in pain.”

**Figure 5:**
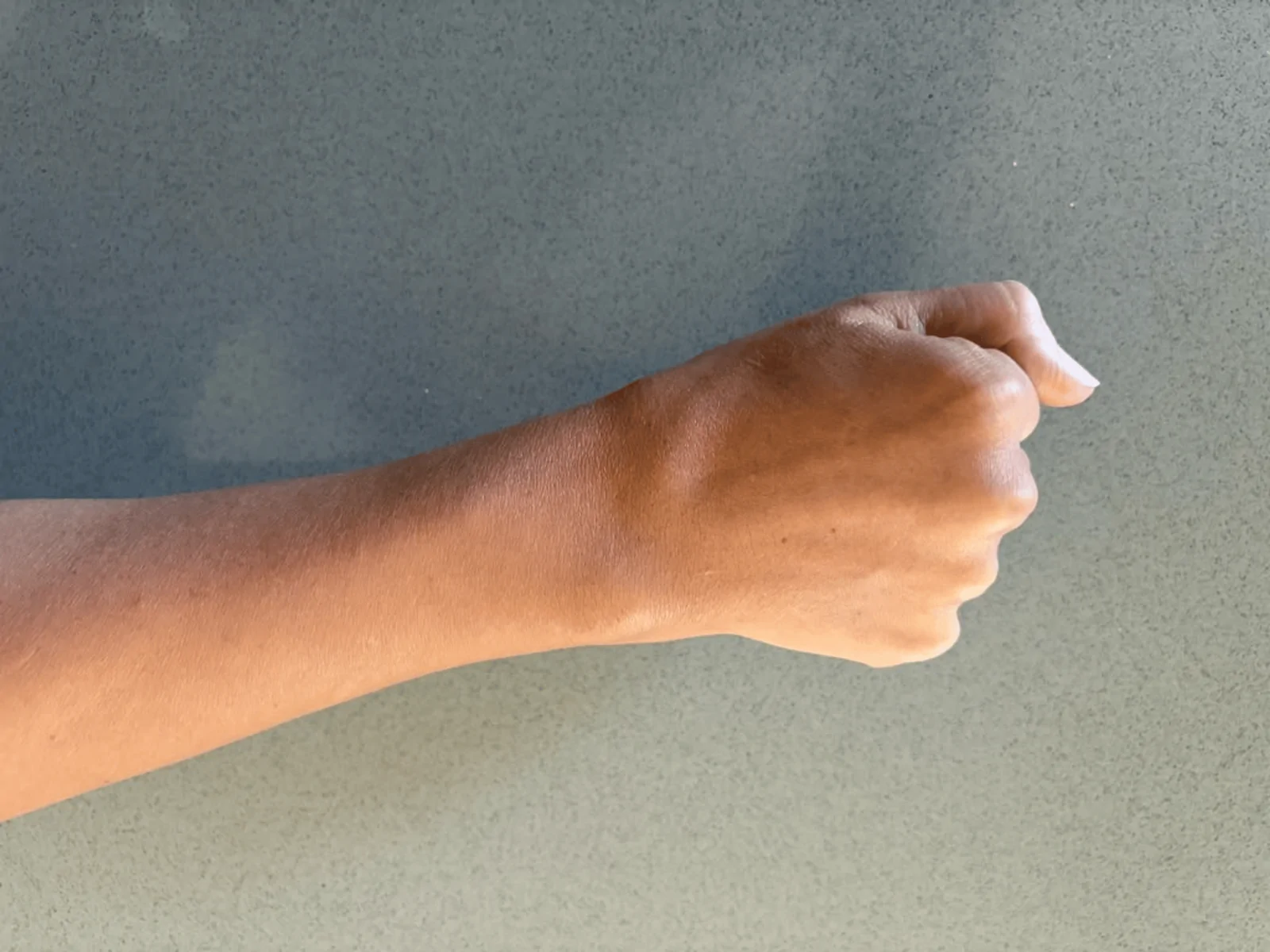
Postoperative wrist appearance 13 days after clip removal

## Discussion

Metal hypersensitivity is an immune-mediated response to exposure to metallic materials and is most commonly associated with allergic contact dermatitis. Nickel is one of the most common metals associated with hypersensitivity reactions and is present in a variety of occupational, consumer, and medical products [3]. Although surgical implants and devices, such as metallic surgical clips, are generally well tolerated, hypersensitivity to implanted metals may present diagnostic challenges, especially when symptoms are systemic or occur beyond the immediate surgical site.

Nickel hypersensitivity is considered a delayed-type hypersensitivity reaction and commonly manifests as localized dermatitis following cutaneous exposure [1]. Although the clinical presentation, diagnostic criteria, and mechanisms of systemic reactions remain poorly established, systemic manifestations have been reported in patients with nickel sensitization [4]. Nonspecific symptoms, such as fatigue, arthralgias, extremity swelling, and musculoskeletal discomfort, may overlap among autoimmune, inflammatory, infectious, and rheumatologic conditions. 

Diagnostic approaches may include a detailed exposure history, LTT, and patch testing. However, no single test is reliable for establishing a correlation between an implanted metal and systemic symptoms [5]. LTT may demonstrate cellular reactivity to specific metals more than others, suggesting that the immune system has been previously sensitized to that material or substance [6].

The association between surgical clip placement and subsequent progressive systemic musculoskeletal symptoms, combined with the patient’s history of nickel-associated allergic dermatitis and nickel reactivity upon LTT, raises the possibility of surgical or implant-related hypersensitivity. The patient’s symptoms developed shortly after placement of the metallic surgical clips and were subsequently attributed to possible post-COVID rheumatoid arthritis; however, all autoimmune testing was negative. The nature of her presentation and the lack of a clear alternative explanation contributed to diagnostic uncertainty. Furthermore, treatments including integrative therapies make it difficult to attribute symptomatic changes to a single intervention. Improvement following platelet-rich fibrin treatment demonstrates that her symptoms were responsive to other interventions and limits the ability to establish a causal relationship with the retained surgical clips.

The delayed identification of the retained surgical clips highlights the importance of considering implanted materials in patients with persistent, unexplained postoperative symptoms. A retrospective history of dermatitis associated with nickel-containing jewelry provided additional context for the patient's reported nickel sensitivity. LTT demonstrated reported reactivity to nickel and iron. However, the clinical significance of these findings in diagnosing metal hypersensitivity remains uncertain. The composition of the surgical clips should also be confirmed through appropriate manufacturer documentation and considered when evaluating unexplained postoperative symptoms.

Although causality cannot be definitively established, the patient reported significant improvement 13 days after removal of the clips. Swelling was reduced, range of motion increased, and pain had lessened. This improvement following removal supports a possible relationship between the nickel-containing clips and the patient’s symptoms. Continued follow-up will be important to determine whether these improvements are sustained.

## Conclusions

This case describes a 36-year-old woman who developed persistent peripheral swelling, arthralgia, and muscle weakness approximately four weeks after bilateral prophylactic mastectomy with immediate reconstruction and placement of nickel-containing surgical clips. Nineteen clips were ultimately removed after the patient became aware of their nickel composition.

This case highlights the importance of considering implanted surgical materials in patients who develop otherwise unexplained inflammatory symptoms following surgery and demonstrates the potential value of documenting the composition of implanted devices. Monitoring the patient's clinical course following removal of the clips will be particularly important in determining whether removal was associated with objective and sustained improvement during outpatient follow-up.
