# Correction: Systemic Inflammatory Symptoms Following Mastectomy With Nickel-Containing Surgical Clips: A Case Report

**DOI:** 10.7759/cureus.c483

**Published:** 2026-09-30

**Authors:** Jasmin Kaur, Anna Kokesh, Frederick Tiesenga

**Affiliations:** 1 Medicine, Windsor University School of Medicine, Cayon, KNA; 2 Medicine, Saint James School of Medicine, Arnos Vale, VCT; 3 General Surgery, West Suburban Medical Center, Oak Park, USA

This article has been corrected to remove the mention of steroid treatment and rheumatoid arthritis diagnosis to ensure that the published case accurately represents the patient's medical history.

